# Effects of Solvent Entropy on Homopolymer Cononsolvency in Binary Solvent Mixtures Predicted by Flory–Huggins Theory

**DOI:** 10.1021/acsmacrolett.6c00080

**Published:** 2026-04-13

**Authors:** Damin Bian, Pengfei Zhang, Baohui Li, Qiang Wang

**Affiliations:** † School of Physics, 12538 Nankai University, No. 94 Weijin Road, Tianjin 90001, P. R. China; ‡ State Key Laboratory of Advanced Fiber Materials, Center for Advanced Low-Dimension Materials, College of Materials Science and Engineering, Donghua University, Shanghai 201620, P. R. China; § School of Biomedical and Chemical Engineering, Colorado State University, 1376 Campus Delivery, Fort Collins, Colorado 80523-1376, United States

## Abstract

Cononsolvency generally refers to the phase separation of a homopolymer solution in mixtures of two miscible good solvents. While commonly observed in systems with a lower critical solution temperature (LCST), it also occurs in systems lacking specific interactions or LCST behavior. Recent coarse-grained polymer models and the classical Flory–Huggins (FH) theory used to study cononsolvency consider only chain connectivity, excluded-volume repulsion, and isotropic van der Waals attractions, with constant nonbonded interaction parameters—making them especially suitable for systems exhibiting an upper critical solution temperature (UCST). However, the role of solvent entropy, particularly the size ratio between the solvent and cosolvent molecules, has been largely overlooked. Extending recent work by one of us (Zhang, P. *Macromolecules* **2024**, *57*, 4298; *ibid.* **2025**, *58*, 2472), we incorporate this entropic effect into the ternary FH theory and find that it significantly broadens the parameter space where cononsolvency occurs in UCST systems.

## Graphical abstract

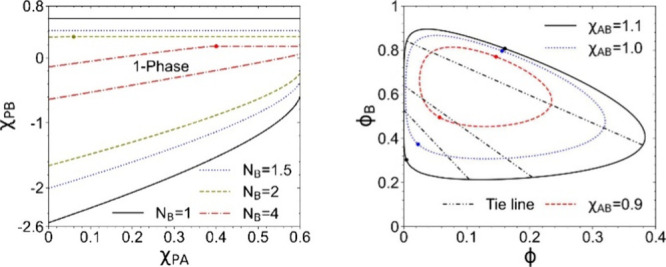

Cononsolvency is an intriguing, experimentally observed phenomenon in which a homopolymer solution undergoes phase separation in a binary solvent mixture, a behavior generally associated with two mutually miscible solvents that are individually good for the polymer.^1^ In single-chain systems, cononsolvency manifests as a sequence of conformational changes from coil to globule to coil, as the solvent composition is varied monotonically. While cononsolvency is commonly observed in systems exhibiting a lower critical solution temperature (LCST), where hydrogen bonding makes the phenomenon highly system-specific, it can also occur in systems lacking such strong interactions or LCST behavior, such as polystyrene in *N*,*N*-dimethylformamide and cyclohexane,^1,2^ poly­(ether imide) in *N*-methyl-2-pyrrolidinone and methylene chloride,^3^ and poly­(methyl methacrylate) in 1-chlorobutane and pentyl acetate (although these are poor solvents for poly­(methyl methacrylate)).^4,5^

Using molecular dynamics simulations of the coarse-grained (CG) Kremer–Grest model including explicit solvent and cosolvent (with the full, instead of the repulsive, Lennard-Jones potential between polymer segments and cosolvent molecules to make the latter a better solvent), Mukherji et al. reproduced the aforementioned conformational change found in experiments and full atomistic simulations.^6^ This was also achieved by Bharadwaj and van der Vegt^7^ using the same approach (but with different parameter values), by Daoulas and co-workers^8^ using fast off-lattice Monte Carlo simulations of a single discrete worm-like chain with the nonbonded potential among all three species described by a third-order polynomial of their local densities (and calculated via a grid), by some of us^9–11^ using lattice Monte Carlo simulations of a self-avoiding-walk chain with only the nearest-neighbor interactions with the two solvents and a Flory-type mean-field theory,^9,11^ and by Meng and co-workers^12^ using fast off-lattice Monte Carlo simulations of a single discrete Gaussian chain with the nonbonded dissipative particle dynamics (DPD) potential among all three species and the self-consistent field calculations of the same model system (where the extended Percus test-particle method^13^ is used to obtain the chain mean-square end-to-end distance at the mean-field level). Moreover, Budkov and co-workers developed an off-lattice mean-field theory that incorporates polymer conformational entropy, local solvent composition, and generic excluded-volume and van der Waals interactions, explaining cononsolvency as an enthalpy–entropy compensation effect.^14^ In a subsequent paper, Budkov and Kolesnikov applied a similar theory (differing mainly in the treatment of attractive interactions) to investigate cononsolvency suppression at high pressures.^15^ These and related theories were reviewed by Budkov and co-workers.^16,17^

As for CG simulations of multichain systems, Mohammadyarloo and Sommer performed MD simulations of the Kremer–Grest model including explicit cosolvent (with the full, instead of the repulsive, Lennard-Jones potential between polymer segments and cosolvent molecules) and implicit solvent at constant pressure for the chains (chosen such that the chains are in the semidilute regime without the cosolvent) and constant chemical potential for the cosolvent molecules.^18^ They found that increasing the cosolvent concentration results in a condensation of the polymer-cosolvent phase accompanied by a sharp drop of the polymer volume, followed by reentry behavior at higher cosolvent concentrations. They also found that the chain conformations follow the semidilute solution scaling and that at higher cosolvent concentrations, an increased excluded volume (due to more adsorbed cosolvent and better solvent quality) swells the chains again; the cosolvent effect is dominated by interchain contacts instead of intrachain cosolvent bridging. Their simulation results for different interaction energies between polymer segments and cosolvent molecules are in good agreement with the prediction of their adsorption-attraction mean-field theory under constant pressure.^18^ Two of us also recently performed lattice Monte Carlo simulations of self- and mutual-avoiding-walk chains with only the nearest-neighbor attraction with cosolvent molecules.^10^ It was found that competition between the system enthalpy and the mixing entropy of binary solvents results in liquid–liquid phase separation (LLPS) of the cosolvent and the cononsolvency. While in single-chain systems the sharing of the localized cosolvent molecules in the LLPS by different chain segments leads to folded chain conformations with its size much smaller than that of the ideal chain, in multichain systems the sharing can be among segments from different chains, which causes chain condensation and hence an average chain size larger than its ideal value.^10^

On the other hand, the classic Flory–Huggins (FH) theory has been applied to describe the (mean-field) phase behavior of such CG polymeric systems. While only binodal curves in the limit of infinite chain length^19^ or spinodal curves^20,21^ (including essentially the same analysis using the random-phase approximation^22–24^ ) of the ternary FH theory were calculated in earlier studies, one of us^25^ recently reported the first complete FH phase diagrams for homopolymer in mixtures of two miscible good solvents. Two of us also compared the binodal curves predicted by the ternary FH theory with lattice Monte Carlo results.^10^ In addition, in the limit of polymer concentration approaching 0, the ternary FH theory gives the effective second virial coefficient of the polymer, which can be used to infer the aforementioned change of chain conformations (at the mean-field level).^12^

A common feature of all of the above CG polymer models and the FH theory is their treatment of interactions, which are limited to chain connectivity, excluded-volume repulsion, and isotropic van der Waals attractions. Here, the nonbonded interaction energy parameters are held constant, making them particularly suitable for systems exhibiting an upper critical solution temperature (UCST), where the FH χ parameters are inversely proportional to the thermodynamic temperature *T* of the system and take (small) non-negative values. As a side note, Dhamankar and Webb recently examined cononsolvency using their newly developed CG model^26^ that incorporates orientation-dependent interactions via a *q*-state Potts framework.^27^

We also note that effects of solvent entropy (i.e., size ratios among the three molecular species), particularly that between the solvent and cosolvent molecules, has not been examined in most of the above studies;^6,8–12,18–21,24–27^ in one of the few exceptions, Bharadwaj and van der Vegt^7^ demonstrated that entropic effects, driven by the size difference between solvent and cosolvent molecules, can lead to preferential adsorption of the larger cosolvent molecules on the polymer chain, even in the complete absence of any attractive interactions. In a previous study by two of us, it was demonstrated for an inhomogeneous system of homopolymer brush in a solvent that properly accounting for the solvent entropy can lead to quantitative agreement between a CG lattice model and experimental measurements without any adjustable parameters.^28^ While the solvent entropy is equivalent to the chain length in the FH theory for a binary system of homopolymer in a solvent, this is not the case for a ternary system including the cosolvent. We therefore extend the recent work by one of us^25,29^ to examine the effects of the size ratio between solvent and cosolvent molecules on the cononsolvency predicted by the ternary FH theory.

The ternary FH theory for homopolymer (P) chains in a mixture of solvent (A) and cosolvent (B) gives the dimensionless Helmholtz free energy of mixing (i.e., in units of *k*_B_ *T* with *k*_B_ being the Boltzmann constant) per volume of an A molecule as

1 $$f=\frac{\phi }{N}\ln\phi +\phi_{A}\ln\phi_{A}+\frac{\phi_{B}}{N_{B}}\ln\phi_{B}+\chi_{\mathrm{PA}}\phi \phi_{A}+\chi_{\mathrm{PB}}\phi \phi_{B}+\chi_{\mathrm{AB}}\phi_{A}\phi_{B}$$

where ϕ, ϕ_A_, and ϕ_B_ are the volume fraction of P, A, and B, respectively, with ϕ + ϕ_A_ + ϕ_B_ = 1, *N* (*N*_B_) is the volume ratio between a P chain (B molecule) and an A molecule, and χ_*ij*_ ≥ 0 is the FH interaction parameter between components *i* (= P, A, B) and *j* ≠ *i* per volume of an A molecule. Clearly, in the case of *N*_B_ = 1, eq 1 reduces to that in the recent work^25,29^ by one of us. For experimental systems, however, it is unlikely that *N*_B_ = 1. Without loss of generality, we take *N*_B_ ≥ 1 hereafter. Taking ϕ and ϕ_B_ as independent variables, we then obtain the dimensionless chemical potential of a polymer segment μ = ∂*f*/∂ϕ, as given by eq 2 in refs 25 and 29 , that of a B molecule μ_B_ = ∂*f*/∂ϕ_B_ = (ln ϕ_B_ + 1)/*N*_B_ – ln (1 – ϕ – ϕ_B_) – 1 + (χ_PB_ – χ_PA_)­ϕ + χ_AB_(1 – ϕ – 2ϕ_B_), which can be compared with eq 3 in refs 25 and 29 , and the dimensionless osmotic pressure

Π=ϕ∂f/∂ϕ+ϕB∂f/∂ϕB−f=(1/N−1)ϕ+(1/NB−1)ϕB−ln⁡(1−ϕ−ϕB)−χPAϕ2−χABϕB2+(χPB−χPA−χAB)ϕϕB,

which can be compared with eq 4 in refs 25 and 29 . We further obtain the equation for the spinodal curve

2 $$p-{\left(\frac{1}{1-\phi -\phi_{B}}+\chi_{\mathrm{PB}}-\chi_{\mathrm{PA}}-\chi_{\mathrm{AB}}\right)}^{2}=0$$

with *p* ≡ [1/*N*ϕ + 1/(1 – ϕ – ϕ_B_) – 2χ_PA_]­[1/*N*_B_ϕ_B_ + 1/(1 – ϕ – ϕ_B_) – 2χ_AB_], which can be compared with eq 11 in ref 25 or eq 5 in ref 29. Finally, we obtain an equation for the critical point (denoted by the subscript “*c*”)

3 $$\frac{{\left(1-a/b\right)}^{3}}{{\left(1-\phi_{c}-\phi_{B,c}\right)}^{2}}+\frac{1}{N_{B}{\phi_{B,c}}^{2}}\frac{a^{3}}{b^{3}}-\frac{1}{N{\phi_{c}}^{2}}=0$$

with *a*/*b* ≡ (*∂* ^2^ *f*/*∂*ϕ^2^)|_*c*_/(∂^2^ *f*/∂ϕ∂ϕ_B_)|_*c*_ as given by eq 14 in ref 25 or eq 7 in ref 29, which can be compared with eq 13 in ref 25 or eq 6 in ref 29; note that eqs 2 and 3 together define the critical point.

At given *N*, *N*_B_, and χ_AB_, the one-phase region ensures that the left-hand-side (LHS) of eq 2 is positive, i.e., χ_PB_ ∈ (χ_PA_ + χ_AB_ – $\sqrt{p}$ – 1/(1 – ϕ – ϕ_B_), χ_PA_ + χ_AB_ + $\sqrt{p}$ – 1/(1 – ϕ – ϕ_B_)), for all possible values of ϕ and ϕ_B_; for each value of χ_PA_, we can therefore find $\chi_{-}^{*}\left(\chi_{\mathrm{PA}}\right)\equiv \underset{\Delta }{\min}\left[\sqrt{p}-1/\left(1-\phi -\phi_{B}\right)\right]$ and $\chi_{+}^{*}\left(\chi_{\mathrm{PA}}\right)\equiv \underset{\Delta }{\min}\left[\sqrt{p}+1/\left(1-\phi -\phi_{B}\right)\right]$ , where the minimization is numerically performed within the triangular domain of ϕ ∈ [0,1], ϕ_B_ ∈ [0,1], and 1 – ϕ – ϕ_B_ ∈ [0,1], and the one-phase region corresponds to χ_PB_ ∈ (χ_PA_ + χ_AB_ – χ_+_^*^, χ_PA_ + χ_AB_ + χ_–_^*^). Note that the binary FH theory for A and B gives its critical point at ${\mathrm{\chi ̅}}_{\mathrm{AB},c}=1/2+1/\sqrt{N_{B}}+1/2N_{B}$ ; we therefore take χ_AB_ < ${\mathrm{\chi ̅}}_{\mathrm{AB},c}$ to ensure the miscibility of A and B. Similarly, the binary FH theory for P and A gives ${\overset{¯}{\chi }}_{PA,c}$ = 1/2 + 1/ $\sqrt{N}$ + 1/2*N*, and that for P and B gives ${\mathrm{\chi ̅}}_{\mathrm{PB},c}=1/2N_{B}+1/\sqrt{NN_{B}}+1/2N$ (and ${\mathrm{\phi ̅}}_{c}=1-{\mathrm{\phi ̅}}_{B,c}=1/\left(1+\sqrt{N/N_{B}}\right)$ ); we therefore take χ_PA_ < ${\overset{¯}{\chi }}_{PA,c}$ and χ_PB_ < ${\overset{¯}{\chi }}_{PB,c}$ to ensure that both A and B are good solvents for P.

The inset of Figure 1a shows the one-phase regions in the χ_PA_–χ_PB_ plane for various χ_AB_ = 0∼1.9 at *N* = 100 and *N*_B_ = 1 (note that ${\overset{¯}{\chi }}_{AB,c}$ = 2 here), which is consistent with Figure 2 of ref 25 (with different ranges of the two axes); Figure 1a itself zooms in on the upper boundary χ_PB_^1+^ ≡ χ_PA_ + χ_AB_ + χ_–_^*^ of these one-phase regions. We see that homopolymer cononsolvency is possible even in mixtures of two miscible good solvents having the same size. The one-phase region, however, is large and occupies most of the parameter space in which we are interested (note that ${\overset{¯}{\chi }}_{PA,c}$ = ${\overset{¯}{\chi }}_{PB,c}$ = 0.605 here). The one-phase region shrinks as χ_AB_ increases, and in most cases (except for small χ_PA_ at χ_AB_ > 1.6) χ_PB_^1+^ coincides with ${\overset{¯}{\chi }}_{PB,c}$ and is independent of χ_PA_ as shown by the right-hand portion of each curve beyond the dot on it. This can be more clearly seen in Figure 1b, where χ_PB_^1+^ at χ_PA_ = 0 and 0.6, as well as ${\overset{¯}{\chi }}_{PB,c}$ , are plotted vs χ_AB_ at *N*_B_ = 1. We also note that when χ_PB_^1+^ coincides with ${\overset{¯}{\chi }}_{PB,c}$ , our numerical calculations indicate ϕ ≈ $\overset{¯}{\phi }$ _*c*_, 1 – ϕ – ϕ_B_ ≈ 0 and ϕ_B_ ≈ $\overset{¯}{\phi }$ _B,*c*_. Similar results are found for various *N*_B_ = 1∼4 at χ_AB_ = 1.12 (note that the smallest ${\overset{¯}{\chi }}_{AB,c}$ here is 1.125, which is for *N*_B_ = 4), as shown in Figure 1c,d. We see that the one-phase region shrinks as *N*_B_ increases, greatly expanding the possible parameter space for homopolymer cononsolvency to occur in mixtures of two miscible good solvents, and that χ_PB_^1+^ coincides with ${\overset{¯}{\chi }}_{PB,c}$ for small *N*_B_ or large χ_PA_. In Section 1 of Supporting Information, we derive the transition value χ_PA_^–^ (χ_PA_^+^) defined as the smallest (largest) value of χ_PA_ at which χ_PB_^1+^ = ${\overset{¯}{\chi }}_{PB,c}$ , ϕ = $\overset{¯}{\phi }$ _*c*_, 1 – ϕ – ϕ_B_ = 0, and ϕ_B_ = $\overset{¯}{\phi }$ _B,*c*_ and given by

4 $$\chi_{\mathrm{PA}}^{\pm }=\chi_{\mathrm{AB}}+\frac{1}{2N}-\frac{1}{2N_{B}}\pm \left(\frac{1}{\sqrt{N}}+\frac{1}{\sqrt{N_{B}}}\right)$$

which are marked by colored dots in Figure 1a,c and are used in Figure S1.

**Figure 1:**
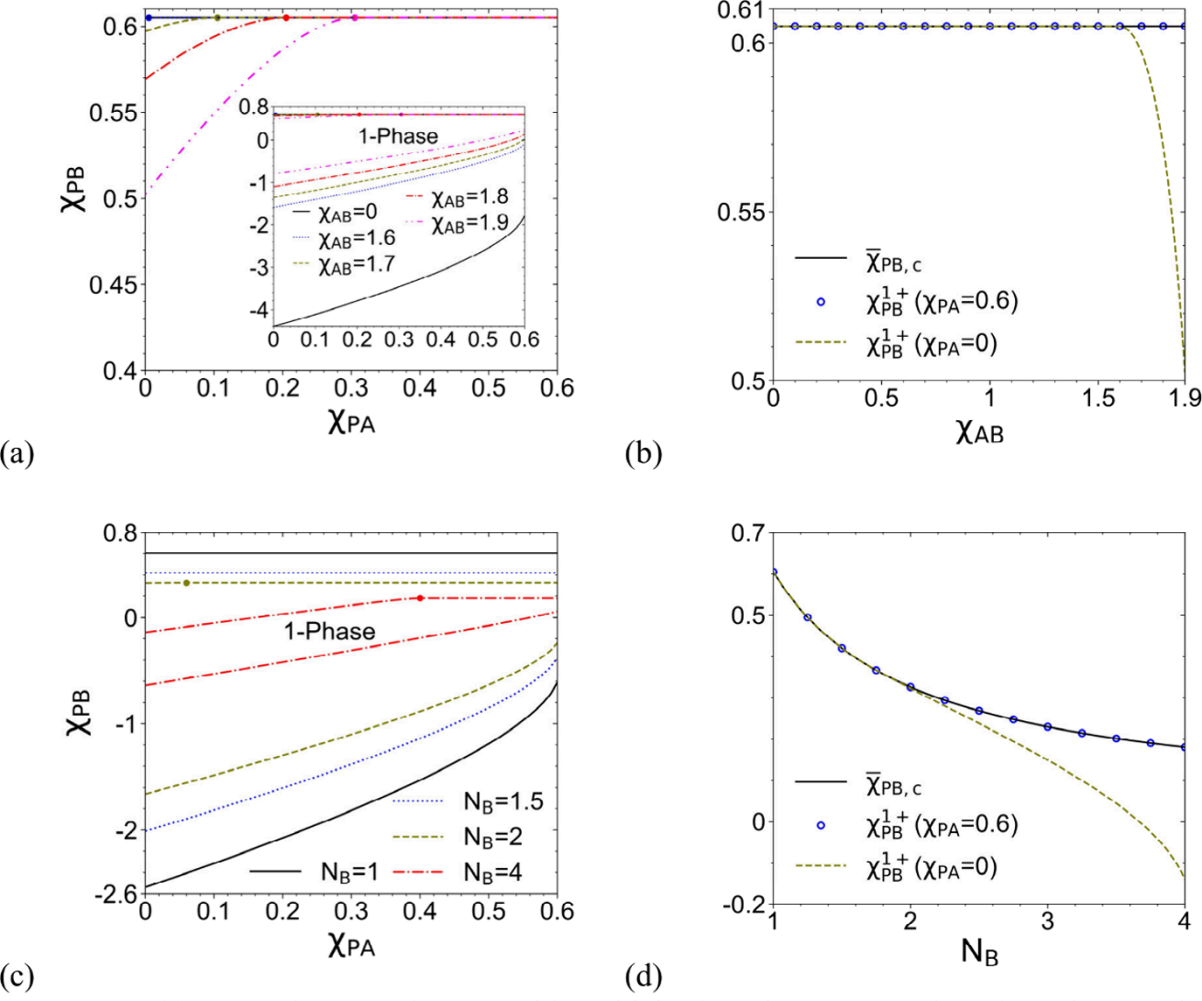
(a) One-phase region, outside which the phase separation (i.e., homopolymer cononsolvency) is possible, and (b) its upper boundary χ_PB_^1+^ at χ_PA_ = 0 and 0.6, as well as the critical point χ*®*_PB,*c*_ given by the binary FH theory for P and B, for various χ_AB_ at *N*_B_ = 1. Part (a) and its inset share the same axes but have different ranges of the vertical axis. Parts (c) and (d) show similar results to parts (a) and (b), respectively, for various *N*_B_ at χ_AB_ = 1.12. In (a) and (c), the dot on a curve having the same color marks the transition value χ_PA_^–^ for that case given by eq 4. *N* = 100 is used in all of the cases. See the main text for details.

To find the binodal curve (i.e., ϕ^I^, ϕ^II^, ϕ_B_^I^ and ϕ_B_^II^) representing the compositions of two different phases I and II at equilibrium given by the homopolymer cononsolvency, we numerically solve the three equations μ^I^ = μ^II^, μ_B_^I^ = μ_B_^II^, and Π^I^=Π^II^ at given *N*, *N*_B_, χ_AB_, χ_PA_, and χ_PB_ outside the one-phase region. With *N* = 100, Figure 2a shows such binodal curves for various χ_PA_ at *N*_B_ = 1, χ_AB_ = 1.9 and χ_PB_ = 0.6, along with the corresponding critical points represented by filled dots. We see that each binodal curve for homopolymer cononsolvency in a mixture of two miscible good solvents forms a closed loop (since there is no phase separation in any of the three binary systems), thus having two critical points; we find these critical points by numerically solving eqs 2 and 3 with different initial guesses. Note that the two critical points merge into one around χ_PA_ = 0.2553 in this case. The area enclosed by the closed loop increases with decreasing χ_PA_ (i.e., increasing difference in the solvent quality between A and B). Similar results are found at *N*_B_ = 2, χ_AB_ = 1.4 and χ_PB_ = 0.32 (note that ${\overset{¯}{\chi }}_{AB,c}$ ≈ 1.457 and ${\overset{¯}{\chi }}_{PB,c}$ ≈ 0.3257 here) shown in Figure 2c, where the two critical points merge into one around χ_PA_ = 0.3061, and at *N*_B_ = 4, χ_AB_ = 1.1 and χ_PB_ = 0.17 (note that ${\overset{¯}{\chi }}_{PB,c}$ = 0.18 here) shown in Figure 2e, where the two critical points merge into one around χ_PA_ = 0.3384. Comparing these three figures (where χ_AB_ and χ_PB_ are close to ${\overset{¯}{\chi }}_{AB,c}$ and ${\overset{¯}{\chi }}_{PB,c}$ , respectively, in each case), we see that the area enclosed by the binodal curve (note the different ranges of the two axes in different figures) increases with increasing *N*_B_, consistent with the effect of varying *N*_B_ on the homopolymer cononsolvency shown in Figure 1a,c. On the other hand, Figure 2b,d,f show the effect of χ_AB_ at *N*_B_ = 1 and χ_PB_ = 0.6, *N*_B_ = 2 and χ_PB_ = 0.32, and *N*_B_ = 4 and χ_PB_ = 0.17, respectively, where χ_PA_ = 0 is used and the two critical points merge into one around χ_AB_ = 1.6800, 1.1426, and 0.8373, respectively. In each figure, we see that the area enclosed by the closed loop increases with increasing χ_AB_.

**Figure 2:**
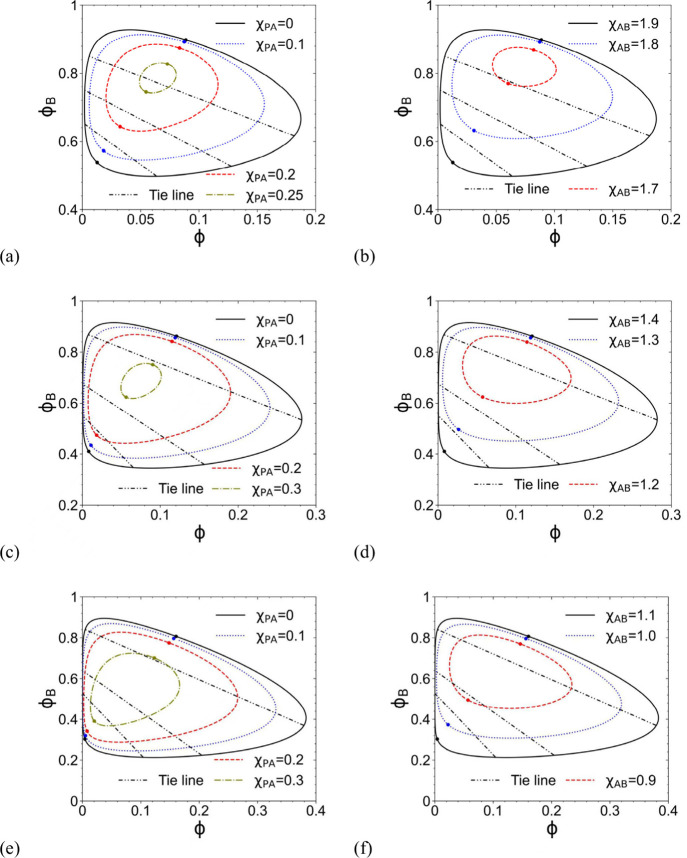
Binodal curves and critical points (represented by filled dots) at (a) *N*_B_ = 1, χ_AB_ = 1.9 and χ_PB_ = 0.6; (b) *N*_B_ = 1, χ_PA_ = 0 and χ_PB_ = 0.6; (c) *N*_B_ = 2, χ_AB_ = 1.4 and χ_PB_ = 0.32; (d) *N*_B_ = 2, χ_PA_ = 0 and χ_PB_ = 0.32; (e) *N*_B_ = 4, χ_AB_ = 1.1 and χ_PB_ = 0.17; and (f) *N*_B_ = 4, χ_PA_ = 0 and χ_PB_ = 0.17. In all parts, *N* = 100 and the black dashed-dotted-dotted lines are the tie lines for the largest binodal loop.

Finally, we note that all tie lines in the ϕ–ϕ_B_ plane have negative slopes *k* < −1; this is consistent with the preferential-interaction mechanism in ref 25, where B is designated as the better solvent and tie lines in the ϕ–ϕ_B_ plane therefore have positive slopes. In contrast, here A is the better solvent for all cases shown in Figure 2; accordingly, the slope of a tie line in the ϕ–ϕ_A_ plane is −(1 + *k*) > 0, indicating enrichment of the better solvent in the P-rich phase. Moreover, increasing *N*_B_ enlarges the binodal loop, indicating stronger composition asymmetry between coexisting phases and therefore stronger preferential enrichment of A in the polymer-rich phase; this is an entropy-driven effect consistent with our FH analysis and prior simulation study^7^ on size-driven preferential adsorption.

In the limit of *N* → ∞, ϕ_*c*_ ∝ *N* ^–1/2^; setting ϕ = ϕ_*c*_ = 0 and ϕ_B_ = ϕ_B,*c*_ in eq 2 then gives a quadratic equation for ϕ_B,*c*_, *Q*ϕ_B,*c*_^2^ – [*Q* + 2­(χ_PA_/*N*_B_ – χ_PB_)]­ϕ_B,*c*_ – (1 – 2χ_PA_)/*N*_B_ = 0, which can be compared with eq 15 in ref 25 or eq 8 in ref 29, with *Q* ≡ 2­(χ_PA_χ_PB_ + χ_PB_χ_AB_ + χ_PA_χ_AB_) – χ_PA_^2^ – χ_PB_^2^ – χ_AB_^2^ as given by eq 9 in ref 29. For cononsolvency to occur in the ternary system of P in two miscible good solvents A and B (i.e., χ_PA_ < 1/2, χ_PB_ < 1/2*N*_B_, and χ_AB_ < ${\overset{¯}{\chi }}_{AB,c}$ ), there must exist two different critical points (thus a positive determinant of the above quadratic equation) satisfying 0 < ϕ_B,*c*_ < 1; this leads to either $\chi_{\mathrm{PB}}<\chi_{\mathrm{PA}}+\chi_{\mathrm{AB}}-1-1/\sqrt{N_{B}}-2\sqrt{\left(1/2-\chi_{\mathrm{PA}}\right)\left({\mathrm{\chi ̅}}_{\mathrm{AB},c}-\chi_{\mathrm{AB}}\right)}$ ,

$$\chi_{\mathrm{PA}}+\chi_{\mathrm{AB}}-1+1/\sqrt{N_{B}}+2\sqrt{\left(1/2-\chi_{\mathrm{PA}}\right)\left(1/2-1/\sqrt{N_{B}}+1/2N_{B}-\chi_{\mathrm{AB}}\right)}<\chi_{\mathrm{PB}}<1/2N_{B}$$

with $\chi_{\mathrm{AB}}<\chi_{\mathrm{PA}}-1/\sqrt{N_{B}}+1/2N_{B}$ (these two conditions can be compared with eqs 15 and 16 in ref 29), or

$$\chi_{\mathrm{PA}}+\chi_{\mathrm{AB}}-1-1/\sqrt{N_{B}}+2\sqrt{\left(1/2-\chi_{\mathrm{PA}}\right)\left({\mathrm{\chi ̅}}_{\mathrm{AB},c}-\chi_{\mathrm{AB}}\right)}<\chi_{\mathrm{PB}}<1/2N_{B}$$

with

$$\chi_{\mathrm{PA}}+1/\sqrt{N_{B}}+1/2N_{B}<\chi_{\mathrm{AB}}<1/2-1/\sqrt{N_{B}}+1/2N_{B}$$

or

$$1/2-1/\sqrt{N_{B}}+1/2N_{B}<\chi_{\mathrm{PA}}+1/\sqrt{N_{B}}+1/2N_{B}<\chi_{\mathrm{AB}}<{\mathrm{\chi ̅}}_{\mathrm{AB},c}$$

(note that replacing the second “<” by “=” here gives χ_PA_^–^ consistent with eq 4). The last condition was missed in ref 29, and we give the detailed derivation of these analytical results in Section 2 of Supporting Information. Figure S2a,b in Supporting Information further show these results at *N*_B_ = 1 (for various χ_AB_) and χ_AB_ = 1.12 (for various *N*_B_), respectively; comparing them with Figure 1a,c, we see that the one-phase region shrinks as *N* increases, expanding the possible parameter space for homopolymer cononsolvency to occur in mixtures of two miscible good solvents.

From eq 1, we see that increasing *N* or *N*_B_ reduces the entropy of mixing and, thereby, destabilizes the homogeneous state. Consequently, the one-phase region shrinks and the binodal loop expands as *N* or *N*_B_ increases. This effect is dominated by *N*_B_, since *N* is typically much larger, making the cosolvent-size dependence far more pronounced. This entropy-driven mechanism underlies the strong sensitivity of cononsolvency to *N*_B_.

To summarize, we have examined, in particular, the effects of the size ratio *N*_B_ ≥ 1 between solvent B and cosolvent A molecules on the homopolymer P cononsolvency in mixtures of the two miscible good solvents predicted by the ternary FH theory. Our study definitively shows that FH theory gives cononsolvency in this system, for which FH theory is applicable, due to the preference of A over B by P. It is found that the one-phase region in the χ_PA_–χ_PB_ plane shrinks as *N*_B_, *N*, or χ_AB_ increase, and that the area enclosed by the closed loop of binodal curves in the ϕ–ϕ_B_ plane increases as *N*_B_, χ_AB_, or |χ_PA_–χ_PB_| increases. We have also derived analytical results for the boundaries of the one-phase region in the χ_PA_–χ_PB_ plane in the limit of *N* → ∞, and those for χ_PA_^±^ given by eq 4. Our results are consistent with and complementary to those recently reported by one of us,^25,29^ and offer concrete guidance for experiments. Because all parameters in our FH theory are experimentally measurable, the predicted shrinkage of the one-phase region and the expansion of the binodal loop with increasing cosolvent size can be directly tested using solvent–cosolvent pairs with tunable molecular volumes. More broadly, the design principles revealed here enable targeted selection of solvent mixtures to probe UCST-type cononsolvency and help distinguish generic entropy-driven behavior from system-specific LCST mechanisms. We expect these predictions to motivate experimental studies that quantitatively examine solvent-entropy effects in mixed-solvent polymer solutions.

## Supplementary Material



## Notes

Derivation of χ_PA_^±^ and figures of our numerical results of δϕ, δϕ_B_, and δχ_PB_ vs δ; and derivation and figures of our analytical results of the one-phase regions in the limit of *N* → ∞ (PDF)
