# Supplementary material for: Effects of Solvent Entropy on Homopolymer Cononsolvency in Binary Solvent Mixtures Predicted by Flory–Huggins Theory
Source: ACS Macro Lett. 2026 Apr 13;15(4):583–8. doi: 10.1021/acsmacrolett.6c00080 (PMC13104173; doi:10.1021/acsmacrolett.6c00080)
Supplement: Supplementary file 1 [file mz6c00080_si_001.pdf]

Supporting Information: Effects of Solvent Entropy on Homopolymer Cononsolvency in Binary Solvent Mixtures Predicted by Flory-Huggins Theory

Damin Bian[1], *Pengfei Zhang*[2], *Baohui Li*[1], and *Qiang Wang*[3,*]

[1]School of Physics, Nankai University,
No. 94 Weijin Rd., Tianjin, 90001, P. R. China
[2]State Key Laboratory of Advanced Fiber Materials, Center for Advanced Low-Dimension Materials, College of Materials Science and Engineering, Donghua University,
Shanghai 201620, P. R. China
[3]School of Biomedical and Chemical Engineering, Colorado State University,
1376 Campus Delivery, Fort Collins, CO 80523-1376, USA

Content:

1. Derivation of $\chi_{PA}^{\pm}$ and figures of our numerical results of $\delta\phi$, $\delta\phi_B$, and $\delta\chi_{PB}$ *vs.* δ.
2. Derivation and figures of our analytical results of the one-phase regions in the limit of $N\rightarrow\infty$.

[*] E-mail: qwang@colostate.edu.

1. Derivation of $\chi_{\mathrm{PA}}^{\pm}$

Here we follow the notation in our main text and derive the transition value χ_{PA}^{-} (χ_{PA}^{+}) defined as the smallest (largest) value of χ_{PA} at which $\chi_{\mathrm{PB}}^{1+} = \bar{\chi}_{\mathrm{PB},c}$, $\phi = \bar{\phi}_c$, $1-\phi-\phi_{\mathrm{B}}=0$, and $\phi_{\mathrm{B}} = \bar{\phi}_{\mathrm{B},c}$. For χ_{PA} close to but smaller than χ_{PA}^{-} (larger than χ_{PA}^{+}) we define a small number $\delta \equiv \chi_{\mathrm{PA}}^{-} - \chi_{\mathrm{PA}} > 0$ ($\delta \equiv \chi_{\mathrm{PA}} - \chi_{\mathrm{PA}}^{+} > 0$); similarly, we have small numbers $\delta\phi \equiv \bar{\phi}_c - \phi$, $\delta\phi_{\mathrm{B}} \equiv \bar{\phi}_{\mathrm{B},c} - \phi_{\mathrm{B}}$, and $\delta\chi_{\mathrm{PB}} = \bar{\chi}_{\mathrm{PB},c} - \chi_{\mathrm{PB}}^{1+}$. Our derivation is based on the assumptions of $\delta\phi = c\delta$, $\delta\phi_{\mathrm{B}} = c_{\mathrm{B}}\delta$, and $\delta\chi_{\mathrm{PB}} = c_{\mathrm{PB}}\delta^2$ in the limit of $\delta \to 0$ with c, c_{B}, and c_{PB} being the proportionality constants; these are supported by our numerical results shown in Fig. S1. Substituting $\chi_{\mathrm{PB}}^{1+} = \bar{\chi}_{\mathrm{PB},c} - \delta\chi_{\mathrm{PB}}$ into $\chi_{\mathrm{PB}}^{1+} \equiv \chi_{\mathrm{PA}} + \chi_{\mathrm{AB}} + \sqrt{p} - 1/(1-\phi-\phi_{\mathrm{B}})$ and solving the resultant quadratic equation for χ_{PA}, we obtain

$$\chi_{\mathrm{PA}} = -1/N_{\mathrm{B}}\phi_{\mathrm{B}} + \chi_{\mathrm{AB}} + \bar{\chi}_{\mathrm{PB},c} - \delta\chi_{\mathrm{PB}} \pm \sqrt{\begin{array}{l}\left(1/N_{\mathrm{B}}\phi_{\mathrm{B}} + 1/N\phi - 2\bar{\chi}_{\mathrm{PB},c}\right)/N_{\mathrm{B}}\phi_{\mathrm{B}} + \left(1/N_{\mathrm{B}}\phi_{\mathrm{B}} + 1/N\phi - 2\bar{\chi}_{\mathrm{PB},c}\right)/\left(1-\phi-\phi_{\mathrm{B}}\right) \\ +2\chi_{\mathrm{AB}}\left(2\bar{\chi}_{\mathrm{PB},c} - 1/N\phi - 1/N_{\mathrm{B}}\phi_{\mathrm{B}}\right) + 2\delta\chi_{\mathrm{PB}}\left[1/N_{\mathrm{B}}\phi_{\mathrm{B}} - 2\chi_{\mathrm{AB}} + 1/\left(1-\phi-\phi_{\mathrm{B}}\right)\right]\end{array}}.$$

Substituting $\chi_{\mathrm{PA}} = \chi_{\mathrm{PA}}^{\pm} \pm \delta$, $\phi_{\mathrm{B}} = \bar{\phi}_{\mathrm{B},c} - c_{\mathrm{B}}\delta$, $\delta\chi_{\mathrm{PB}} = c_{\mathrm{PB}}\delta^2$, and $\phi = \bar{\phi}_c - c\delta$ into it, we finally obtain at the leading order Eq. (4) in our main text, where we have used $\bar{\chi}_{\mathrm{PB},c} = 1/2N_{\mathrm{B}} + 1/\sqrt{NN_{\mathrm{B}}} + 1/2N$ and $1-\bar{\phi}_{\mathrm{B},c} = \bar{\phi}_c = 1/\left(1+\sqrt{N/N_{\mathrm{B}}}\right)$; note that $2\bar{\chi}_{\mathrm{PB},c} - 1/N\bar{\phi}_c - 1/N_{\mathrm{B}}\bar{\phi}_{\mathrm{B},c} = 0$ and that at the leading order $\left(1/N_{\mathrm{B}}\phi_{\mathrm{B}} + 1/N\phi - 2\bar{\chi}_{\mathrm{PB},c}\right)/\left(1-\phi-\phi_{\mathrm{B}}\right) = \left(\sqrt{N} + \sqrt{N_{\mathrm{B}}}\right)^2/NN_{\mathrm{B}}$ under the above assumptions.

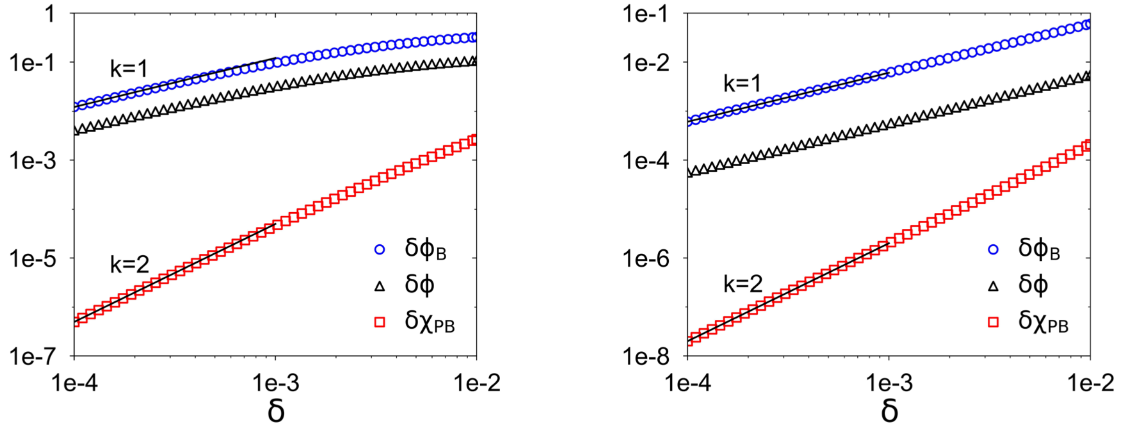

(a) (b)

Figure S1: Logarithmic plots of our numerical results of $\delta\phi$, $\delta\phi_{\mathrm{B}}$, and $\delta\chi_{\mathrm{PB}}$ *vs.* δ defined in Sec. 1 at (a) $\chi_{\mathrm{AB}}=1.12$ (where $\delta \equiv \chi_{\mathrm{PA}}^{-} - \chi_{\mathrm{PA}}$ with $\chi_{\mathrm{PA}}^{-} = 0.4$ given by Eq. (4) in the main text) and (b) $\chi_{\mathrm{AB}}=0$ (where $\delta \equiv \chi_{\mathrm{PA}} - \chi_{\mathrm{PA}}^{+}$ with $\chi_{\mathrm{PA}}^{+} = 0.48$ given by Eq. (4) in the main text), where the k-values give the slopes of the corresponding straight lines, and $N_{\mathrm{B}}=4$ and $N=100$ are used in both cases. See Sec. 1 for more details.

2. Analytical results of the one-phase regions in the limit of $N\to\infty$

Here we follow our notation in the main text and derive the analytical results of the one-phase regions in the limit of $N\to\infty$, where $\phi_c \propto N^{-1/2}$. Setting $\phi=\phi_c=0$ and $\phi_B=\phi_{B,c}$ in Eq. (2) in the main text then gives a quadratic equation $a\phi_{B,c}{}^2 + b\phi_{B,c} + c = 0$ with $a \equiv \chi_{PA}{}^2 + \chi_{PB}{}^2 + \chi_{AB}{}^2 - 2(\chi_{PA}\chi_{PB} + \chi_{PA}\chi_{AB} + \chi_{PB}\chi_{AB})$, $b \equiv -a + 2(\chi_{PA}/N_B - \chi_{PB})$, and $c \equiv (1-2\chi_{PA})/N_B$. By setting its determinant $\Delta \equiv b^2 - 4ac$ to 0 and solving for χ_{AB}, we obtain four characteristic χ_{AB}-values, *i.e.*,

$$\chi_{AB,1} = \chi_{PA} + \chi_{PB} - \sqrt{2/N_B}\sqrt{1-\chi_{PA}-\chi_{PB}N_B(1-2\chi_{PA}) + \sqrt{(1-2\chi_{PA})(1-2\chi_{PB}N_B)}},$$

$$\chi_{AB,2} = \chi_{PA} + \chi_{PB} - \sqrt{2/N_B}\sqrt{1-\chi_{PA}-\chi_{PB}N_B(1-2\chi_{PA}) - \sqrt{(1-2\chi_{PA})(1-2\chi_{PB}N_B)}},$$

$$\chi_{AB,3} = \chi_{PA} + \chi_{PB} + \sqrt{2/N_B}\sqrt{1-\chi_{PA}-\chi_{PB}N_B(1-2\chi_{PA}) - \sqrt{(1-2\chi_{PA})(1-2\chi_{PB}N_B)}},$$

$$\chi_{AB,4} = \chi_{PA} + \chi_{PB} + \sqrt{2/N_B}\sqrt{1-\chi_{PA}-\chi_{PB}N_B(1-2\chi_{PA}) + \sqrt{(1-2\chi_{PA})(1-2\chi_{PB}N_B)}},$$

which are all real for the ternary system of P in two miscible good solvents A and B (*i.e.*, $\chi_{PA}<1/2$, $\chi_{PB} < 1/2N_B$, and $\chi_{AB} < \bar{\chi}_{AB,c} = \left(1+1/\sqrt{N_B}\right)^2/2$), with $\chi_{AB,1}<\chi_{AB,2}<\chi_{AB,3}<\chi_{AB,4}$. At given χ_{PA}, χ_{PB}, and N_B, these χ_{AB}-values then partition the entire χ_{AB} axis into distinct regimes. For cononsolvency to occur in the ternary system, there must exist two different critical points (thus $\Delta>0$). In each χ_{AB}-regime where $\Delta>0$, we then calculate $\phi_{B,c} = \left(-b \pm \sqrt{\Delta}\right)/2a$, and a physical critical point requires $0<\phi_{B,c}<1$. In particular, we find that the cononsolvency occurs when $\chi_{AB}<\chi_{AB,1}$ or $\chi_{AB,4} < \chi_{AB} < \bar{\chi}_{AB,c}$.

Equivalently, by setting Δ to 0 and solving for χ_{PB}, we obtain four characteristic χ_{PB}-values, *i.e.*,

$$\chi_{PB,1} = \chi_{PA} + \chi_{AB} - 1 - 1/\sqrt{N_B} - 2\sqrt{(1/2-\chi_{PA})(\bar{\chi}_{AB,c} - \chi_{AB})},$$

$$\chi_{PB,2} = \chi_{PA} + \chi_{AB} - 1 - 1/\sqrt{N_B} + 2\sqrt{(1/2-\chi_{PA})(\bar{\chi}_{AB,c} - \chi_{AB})},$$

$$\chi_{PB,3} = \chi_{PA} + \chi_{AB} - 1 + 1/\sqrt{N_B} - 2\sqrt{(1/2-\chi_{PA})(\bar{\chi}_{AB,c} - 2/\sqrt{N_B} - \chi_{AB})},$$

$$\chi_{PB,4} = \chi_{PA} + \chi_{AB} - 1 + 1/\sqrt{N_B} + 2\sqrt{(1/2-\chi_{PA})(\bar{\chi}_{AB,c} - 2/\sqrt{N_B} - \chi_{AB})}.$$

For the above ternary system, we note that when $\chi_{AB} < \bar{\chi}_{AB,c} - 2/\sqrt{N_B}$, all these χ_{PB}-values are real and partition the entire χ_{PB} axis into five distinct regimes, with $\chi_{PB,1}<\chi_{PB,2}$, $\chi_{PB,3}<\chi_{PB,4}$, and $\chi_{PB,1}<\chi_{PB,3}$; in this case, we find that the cononsolvency occurs when $\chi_{PB}<\chi_{PB,1}$, $\chi_{PB,4} < \chi_{PB} < 1/2N_B$ with $\chi_{AB} < \chi_{PA} + 1/2N_B - 1/\sqrt{N_B}$, or $\chi_{PB,2} < \chi_{PB} < 1/2N_B$ with $\chi_{PA} + 1/2N_B + 1/\sqrt{N_B} < \chi_{AB} < \bar{\chi}_{AB,c} - 2/\sqrt{N_B}$. On the other hand, when $\bar{\chi}_{AB,c} - 2/\sqrt{N_B} < \chi_{AB} < \bar{\chi}_{AB,c}$, only $\chi_{PB,1}$ and $\chi_{PB,2}>\chi_{PB,1}$ are real and they partition the χ_{PB} axis into three regimes; in this case, the cononsolvency occurs when $\chi_{PB}<\chi_{PB,1}$ or $\chi_{PB,2} < \chi_{PB} < 1/2N_B$ with

$\chi_{PA} + 1/2N_B + 1/\sqrt{N_B} < \chi_{AB} < \bar{\chi}_{AB,c}$. Combining these results leads to the cononsolvency condition given in our main text.

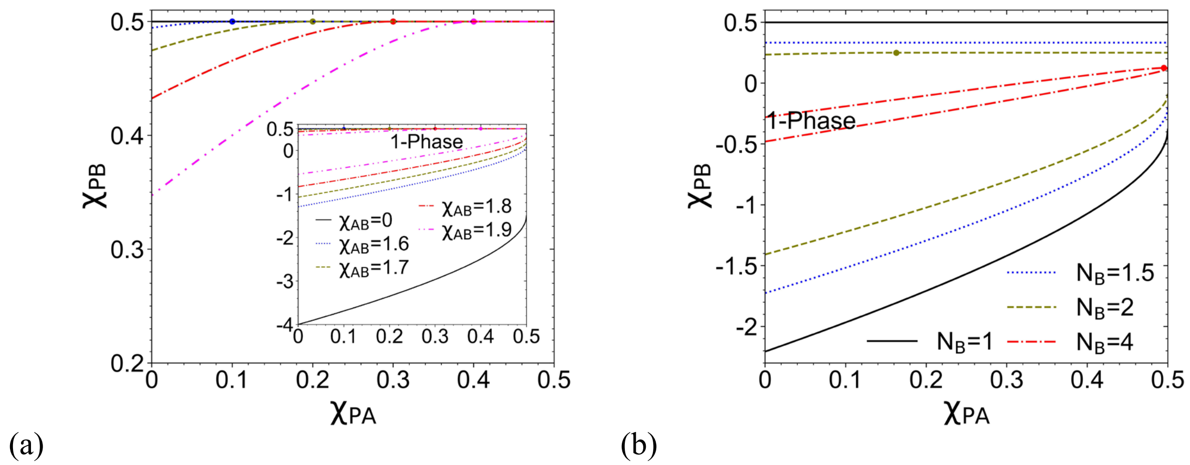

Figure S2: One-phase regions, outside which the phase separation (*i.e.*, homopolymer cononsolvency) is possible, for (a) various χ_{AB} at N_B=1 and (b) various N_B at χ_{AB}=1.12, both in the limit of $N \to \infty$. The dot on a curve having the same color marks the transition value χ_{PA}^{-} for that case given by Eq. (4) in the main text. See the main text for details.
